# Kidney Transplantation in Western Balkans: A Regional Blueprint for Access, Capacity, and Equity

**DOI:** 10.3389/ti.2026.15952

**Published:** 2026-03-13

**Authors:** Elvana Rista, Goce Spasovski, Damir Rebic, Mirjana Lausevic, Danilo Radunovic, Vjollca Godanci, Ariana Strakosha, Alma Idrizi, Kristi Saliaj, Emin Baris Akin, Jelena Stojanovic, Fernanda Ortiz, Carmen Lefaucheur, Luciano Potena, Efstratios Chatzixiros, Jamil Azzi, Devi Mey

**Affiliations:** 1 European University of Tirana, Tirana, Albania; 2 Department of Nephrology, Dialysis and Transplantation, Hygeia Hospital Tirana, Tirana, Albania; 3 Sts. Cyril and Methodius, University of Skopje, Skopje, North Macedonia; 4 Clinical Center University of Sarajevo, Sarajevo, Bosnia and Herzegovina; 5 Medical Faculty, University of Belgrade, Belgrade, Serbia; 6 University Clinical Centre of Serbia, Belgrade, Serbia; 7 Clinical Center of Montenegro, Podgorica, Montenegro; 8 Nephrology Clinic, UCCK University Clinical Centre of Kosova, Pristina, Kosovo; 9 University Hospital Center “Mother Teresa”, Tirana, Albania; 10 Demiroglu Bilim University Florence Nightingale Hospital, Istanbul, Türkiye; 11 Great Ormond Street Hospital for Children NHS Foundation Trust, London, United Kingdom; 12 Abdominal Unit, Nephrology, Helsinki University Hospital, Helsinki, Finland; 13 Saint Louis Hospital, Assistance Publique-Hopiteux de Paris and Université Paris Cité, Paris, France; 14 IRCCS University Hospital of Bologna Sant’ Orsola Polyclinic, Bologna, Italy; 15 Medicines-Health Products Policies and Standards Department, World Health Organization (WHO), Geneva, Switzerland; 16 Brigham and Women’s Hospital, Harvard Medical School, Boston, MA, United States; 17 The European Society for Organ Transplantation, Amsterdam, Netherlands

**Keywords:** blueprint, equity, health systems, kidney transplantation, Western Balkans

## Abstract

There is no medical field where the impact of medical evolution is more palpable than in kidney transplantation. The pioneers of this procedure, 70 years ago, laid out the foundation for organ transplantation in general and kidney transplantation in particular. Despite the incredible advancements that have been made since, huge differences exist worldwide in terms of access, equity and quality of care. Nowhere are these disparities more prominent than in developing countries with limited resources, underfunded healthcare systems and transplantation infrastructures, particularly the Western Balkans. This position paper delineates the biggest barriers hindering the development of kidney transplantation in the Western Balkans, put forth and agreed upon by a group of regional experts on the field, based on the Modified Delphi Method. Limitations in training, infrastructure, restrictive and outdated legislative practices, lack of a centralized coordination network and fragmented regional collaboration, emerged as the principal challenges. Endorsed by European Society for Organ Transplantation (ESOT), this paper outlines a pragmatic and practical framework to overcome these obstacles, towards building robust and sustainable transplantation programs that ensure high-quality and equitable access to kidney transplantation, for all patients in this region.

## Introduction

Chronic kidney disease (CKD) represents a growing public health challenge, associated with significant morbidity and mortality rates and substantial implications across public healthcare systems. Affecting over 850 million people worldwide, recent data places CKD as the 7th leading cause of death globally and has shown a sharp rise in disability-adjusted life years (DALYs) and years of life lost (YLLs), particularly in low- and middle-income countries [1–4].

In light of this data, in 2024 the International Society of Nephrology (ISN), American Society of Nephrology (ASN) and European Renal Association (ERA) in a joint statement proposed to include CKD in the WHO’s list of major non-communicable diseases, due to its significant contribution in premature cardiovascular-related deaths, expecting it to become the fifth leading cause of death worldwide by 2040 [5].

Kidney transplantation is widely recognized as the gold standard, but dialysis remains the cornerstone of CKD management. Nevertheless, evidence consistently shows transplantation to be associated with superior survival rates, improved quality of life, psychosocial wellbeing, social and workforce reintegration, as well as higher employment rates, compared to dialysis patients [6–9]. Moreover, from a health economics standpoint, transplantation proves to be more cost-effective in the long run. In Europe, the annual cost per patient for dialysis is estimated to vary between €50,000 to €90,000, while transplantation, despite higher upfront costs, yields lower long-term expenditures not only due to the ommited dialysis cost, but also to reduced hospitalization and complication rates [10, 11].

Between 10% and 30% of patients on waiting lists die before receiving a graft, reflecting persistent organ shortages and systemic inefficiencies (ERA Registry 2022; EDQM Newsletter Transplant 2024) [12, 13]. Despite these clear advantages, disparities in access, limited organ availability, inefficiencies and lack of resources across healthcare systems, legislative and cultural barrierscontinue to hinder its full potential [14]. A region facing these challenges is the Western Balkans (Albania, Bosnia and Herzegovina, Kosovo, Montenegro, North Macedonia, Serbia), where equity and access to kidney transplantation remains markedly lower than in the rest of the continent [9, 15, 16].

In this context, on May 24 -25, 2025 an important conference aiming to provide a better understanding of the current landscape and the challenges in kidney transplantation in the Western Balkans was held, in Tirana, Albania. Transplantation Without Borders: Balkan Initiative supported by the European Society for Organ Transplantation (ESOT), brought together regional experts and international leaders in the field, to address the structural, systemic and legislative barriers currently being faced in the Western Balkans. Additionally, it set to develop a blueprint on the steps that are required to move toward robust, active national programs and a unified regional collaboration to ensure faster and more equitable access, as well as better quality of care for patients in the region, in line with the ESOT Manifesto aiming to tackle inequality in organ transplantation [17].

This position paper summarizes the data on the current landscape of kidney transplantation in the Western Balkans, and outlines an actionable, collaborative regional strategy to improve outcomes through harmonized legislation, optimized living and deceased donor programs, and shared capacity- building frameworks.

## Materials and Methods

### Study Design

**FIGURE 1:**
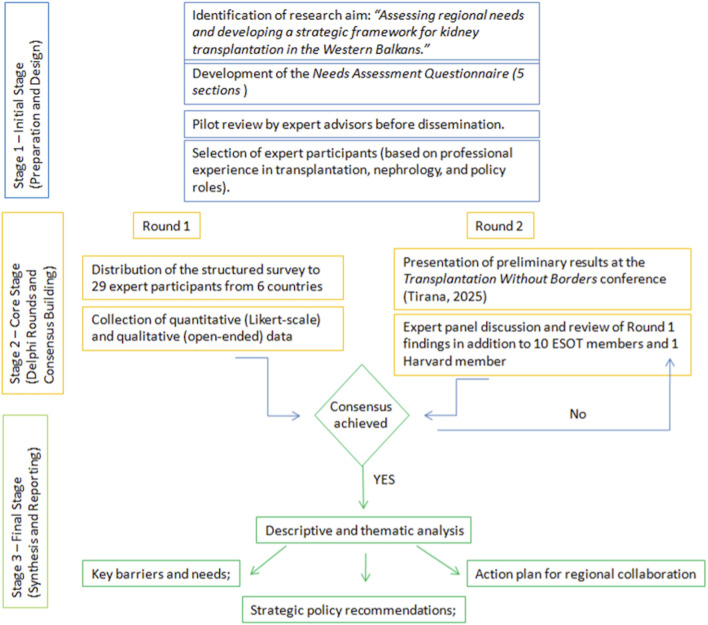
Flow diagram illustrating the Modified Delphi Method applied to the Western Balkans kidney transplantation needs assessment.

This study employed a Modified Delphi Method, integrating quantitative and qualitative data collection to identify regional needs, barriers, and priorities for advancing kidney transplantation in the Western Balkans. The Delphi approach is designed to gather expert consensus through iterative rounds of structured feedback [18]. In this study, a modified format was adopted, consisting of a structured needs- assessment survey and expert roundtable discussions conducted during the regional conference “Transplantation Without Borders: Balkan Initiative” (Tirana, 2025) (Figure 1).

The objective of this methodological approach was to facilitate systematic consensus among nephrologists, transplant surgeons, immunologists, coordinators, and policymakers representing Western Balkan countries and regional model country (Croatia) on the current landscape and future strategic approach for kidney transplantation in the region.

### Delphi Procedure

The first round was conducted through a structured online questionnaire, developed by EKITA-ESOT, distributed to transplant experts. The survey included five themes of questions:

1. Demographic and institutional characteristics;
2. Current national transplantation activity and infrastructure;
3. Barriers related to legislation, organization, training, and immunology;
4. Pharmacological and coordination system limitations;
5. Strategic priorities and feasible short-term actions.

The questionnaire combined closed questions, Likert-scale items assessing perceived importance or limitation, and open-ended questions that allowed for qualitative elaboration. Responses were analyzed descriptively to identify the most frequently cited barriers and priority areas.

The second round took place during the in-person conference sessions, where preliminary survey results were presented. Participants engaged in moderated roundtable discussions structured around five thematic domains: legislation, capacity building, infrastructure, pharmacological access, and coordination systems. Consensus was defined *a priori* as ≥75% agreement among participants on a given recommendation or priority area (Supplementary Table 1). Revised consensus statements were integrated into the final regional position framework.

### Post-Conference Validation and Country-Specific Data Collection

Following the completion of the two Delphi rounds and the consensus meeting, an additional post- conference validation phase was conducted to complement and refine the findings. During this stage, national experts from each participating Western Balkan country were invited to provide updated and country-specific information through structured online communication.

Between May and October 2025, experts from Western Balkans submitted detailed written feedback via email correspondence coordinated by the study team. Each expert provided validated information on key indicators, including population data, dialysis prevalence, number of active transplant centers, annual kidney-transplantation activity, legal framework, coordination systems, and existing training infrastructure.

### Data Analysis

Qualitative responses were subjected to thematic analysis using an inductive approach following Braun and Clarke’s six-phase framework. Emerging categories were grouped into overarching themes corresponding to the main barriers and proposed solutions.

Country-specific expert reports in the post-conference validation were analyzed descriptively and merged into a unified regional dataset.

Raw country data were range normalized to a 0–1 scale. For each country, five indicators were compiled (CKD prevalence, dialysis burden, annual transplant rate, number of active centers, and deceased-donor program status) inverted so higher scores indicate greater capacity. Normalized values were plotted in Excel as a radar chart to visualize cross-country readiness.

### Outcome

The Modified Delphi process resulted in a consensus-based regional framework outlining actionable recommendations for improving kidney transplantation across the Western Balkans. The findings serve as the foundation for the regional position statement “Transplantation without Borders: Balkan Initiative.

## Results

### Participant Characteristics

A total of 29 experts participated in the Modified Delphi survey, representing six Western Balkan countries and Croatia. The panel comprised 66% female and 34% male participants, with a mean age of 45.9 ± 10.2 years (range 28–69 years). Most respondents were nephrologists involved in kidney transplantation (72%), while others included transplant surgeons, coordinators, immunologists, and patient organization representatives (Table 1).

**TABLE 1:** Demographic and professional characteristics of the 29 experts who participated in the Modified Delphi survey on kidney transplantation in the Western Balkans.

Characteristic	Category/description	N (%) (Total 29)
Gender	Male	10 (34%)
	Female	19 (66%)
Age (years)	Mean ± SD (range)	45.9 ± 10.2 (28–69)
Country of affiliation	Albania	8 (27.6%)
	Bosnia and Herzegovina	4 (13.8%)
	Kosovo	2 (6.9%)
	Montenegro	3 (10.3%)
	North Macedonia	5 (17.2%)
	Serbia	3 (10.3%)
	Croatia	4 (13.7%)
Professional role	Nephrologist	21 (72%)
	Transplant surgeon	3 (10%)
	Transplant coordinator	1 (3%)
	Laboratory/histocompatibility Specialist	1 (3%)
	Patient representative/ Organization	2 (7%)
	Other (resident/Fellow)	1 (3%)
Institution type	University hospital/Clinical center	23 (79%)
	National/ Academic/Research institute	6 (21%)

### Qualitative Analysis

Analysis of open-ended survey responses and expert discussions revealed five related themes reflecting shared regional challenges and strategic priorities for kidney transplantation in the Western Balkans. These themes, derived through the Modified Delphi process, highlight the experts’ collective perspective on operational barriers, emphasized the need for harmonized legislative frameworks, improved infrastructure, and immunological capacity, strengthened workforce training and improved access to new pharmacological options across the region. A summary of these thematic domains and representative insights are presented in Table 2.

**TABLE 2:** Summary of qualitative themes identified through the Modified Delphi process.

Theme	Description	Representative expert insights
Legislative and regulatory fragmentation	Variation in national frameworks limits living, deceased and kidney paired donation programs	“Our legal framework exists, but it’s fragmented and not fully operational — we need harmonized regional standards.”(Expert, Montenegro)
Immunological and pharmacological capacity gaps	Lack of advanced immunological testing and new therapeutic agents	“The need to outsource advanced immunological testing abroad, delays transplantation access to our patients.”(Expert, Albania)
Workforce and training needs	Shortage of transplant coordinators, surgeons, and nephrologists; lack of structured professional training	“We need not only more specialists but also a defined career path in transplantation.”(Expert, Bosnia and Herzegovina)
Limited infrastructure and coordination systems	Outdated or limited infrastructure and centralized coordination systems	“Even when we have trained specialists, the absence of a dedicated transplant center and staff significantly limits our transplantation services.” (Expert, North Macedonia)
Regional cooperation	Absence of unified national registries, regional standardized protocols, and coordination systems limits the scope of transplantation activity	“In addition to empowering national programs, fostering regional collaboration is crucial in improving transplantation services in the region.” (Expert, Serbia)

### Country Specific Reports and Expert Feedback

**FIGURE 2:**
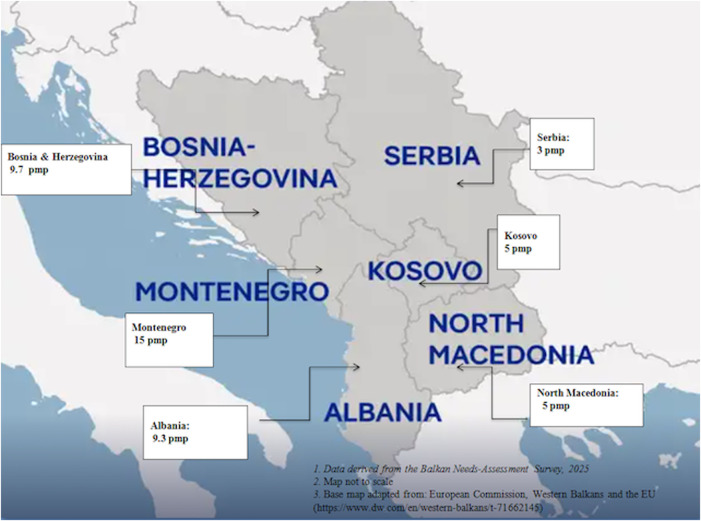
Kidney transplantation indicators in the Western Balkans (2025).

**FIGURE 3:**
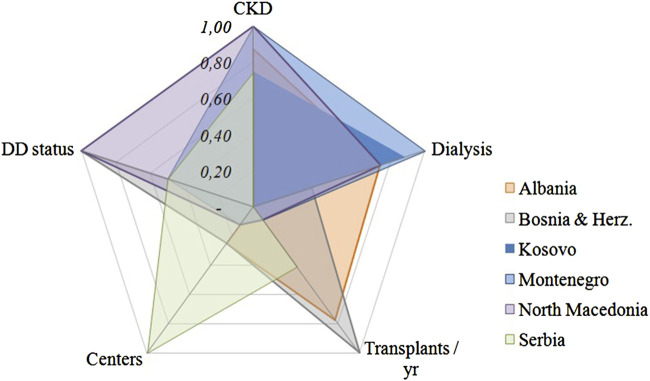
Radar chart comparing kidney-transplantation readiness across five domains in six Western Balkan countries. Indicators were normalized to 0–1 (higher = greater capacity); CKD prevalence and dialysis burden were inverted. Data: Delphi survey and post-conference expert validation (2025).

Experts from each participating country provided quantitative and contextual updates on national kidney-transplantation indicators. The findings are summarized in Figure 2 and Table 3, providing a comparative overview of population metrics, dialysis and transplantation activity, program structure, and the main challenges identified across the Western Balkans. Figure 2 shows that pmp rates remain very low across all active transplant centers in the Western Balkans. The country-level radar plots were generated to compare transplantation system readiness across five domains (CKD prevalence, dialysis burden, annual transplants, active centers, and deceased-donor status). Raw values were standardized to a 0–1 scale using range normalization, with limitation indicators (CKD, dialysis) inverted so that higher values uniformly indicate greater capacity. The charts visualize relative strengths and gaps across countries Figure 3.

**TABLE 3:** Country-specific summary of kidney transplantation indicators.

Country	Population (millions)	CKD Prevalence (%)	Dialysis patients (n)	Kidney transplants (total/annual)	Deceased donor program	Active centers (n)	PMP (per million population)
Albania	2.7	10.5	1800	400 total/ 25–30 per year	No (only living donor)	2 (both in Tirana)	9.3
Bosnia and Herzegovina	3.5	14	3,800	990 total/ 30–35 per year	Yes (limited)	2 (Sarajevo, Tuzla)	9.7
Kosovo	1.6	11	1,044	150 total/ ∼8 per year (abroad)	No	0	5
Montenegro	0.63	10	495	200 total/ 10–12 per year	Inactive	1 (Podgorica)	15
North Macedonia	1.79	10%	1792	512 total/ 10 per year	Yes	1	5
Serbia	6.6	≈10–11	5,500	≈850 living patients/ ≈17 per year	Yes (limited activity)	8	3

#### Albania

“*The need to outsource advanced immunological testing abroad, delays transplantation access to our patients*.” (Expert, Albania)

Albania has a population of approximately 2.7 million people, with a median age of 37.9 years. It is estimated that over 10.5% of the population is affected by CKD, with more than 200,000 people living with some stage of CKD. Among them, 1800 patients with end-stage kidney disease (ESKD) are currently receiving renal replacement therapy. Around 1,400 patients (75.9%) undergo hemodialysis with an estimated annual incidence increase between 5%-7%. Hemodialysis services are distributed across 14 active units nationwide. Thirty-five patients (1.9%) are on peritoneal dialysis (PD). A total of 400 patients (22.2%) have received a kidney transplant, with an average of 25–30 new cases conducted annually.

The basic legal framework on transplantation in Albania began to take shape in 1997, when the first legislation regulating organ transplantation was passed. A decade later, in 2007 the first kidney transplant in a public institution was performed as part of an international collaboration with the University Hospital of Bari supported by the INTERREG III Project. This initiative aimed to develop the necessary infrastructure and provide adequate training to healthcare providers involved in kidney transplantation. In 2011, the adoption of the National Law on Organs, Tissues and Cells by the Albanian Parliament paved the way for the formal introduction of kidney transplantation as a therapeutic modality for patients with ESKD.

However, despite the progress made, numerous challenges persist. At present, only living donor kidney transplantation (LDKT) is performed in Albania. Two active transplant centers, both located in Tirana, carry out all procedures. In 2024, 26 living donor kidney transplants were performed, corresponding to a rate of 9.3 per million population (pmp). There is currently no active deceased donor kidney transplantation (DDKT) program in the country.

Our survey identified insufficient training, infrastructure, and regulatory barriers as the main challenges limiting the development of kidney transplantation in the country. There are no formal training programs for transplant surgeons or nephrologists, and most healthcare professionals have obtained only short-term training abroad. Surgical procedures are performed by hybrid teams, combining local nephrologists and anesthesiologists with visiting surgeons from Turkey.

Significant infrastructural constraints, particularly in immunological evaluation, were also reported. While basic pre-transplant testing is performed locally, advanced assays are outsourced abroad due to limited equipment and capacity. Although standard immunosuppressive agents are available through public reimbursement, access to newer therapies for rejection, desensitization, and drug-resistant infections remains limited.

Participants emphasized the need to update national transplantation legislation, particularly to include paired donation and strengthen frameworks for both living and deceased donation. Finally, the absence of a centralized coordination system and dedicated transplant coordinators was identified as a critical barrier to improving inter-institutional collaboration and overall program efficiency.

#### Bosnia and Herzegovina

“*We need not only more specialists but also a defined career path in transplantation*.” (Expert, Bosnia and Herzegovina)

Bosnia and Herzegovina, with a population of approximately 3.5 million and a median age of 49.7 years, faces a substantial burden of CKD, affecting an estimated 14% of the population, over 400,000 individuals. Among them, around 3,800 patients are living with ESKD and currently receive renal replacement therapy. Approximately 2,800 patients are treated with hemodialysis or peritoneal dialysis, with an annual incidence increase of 5.6%. Dialysis services are provided through 26 active centers nationwide. Sixty-eight patients are maintained on PD. To date, a total of 990 kidney transplants have been performed in the country, with an average of 30–35 new transplants per year.

The first kidney transplant in Bosnia and Herzegovina was performed in Sarajevo in 1974, marking the second in the former Yugoslavia. This early achievement established a solid foundation with trained professionals and a functioning transplant infrastructure. However, the Bosnian war interrupted program development, halting progress after nearly 300 kidney transplants had been performed. Following the war’s end in 1995, transplantation activities resumed under the pre-existing National Law on Organ and Tissue Transplantation at three centers, Sarajevo, Tuzla, and Banja Luka, though national activity remained low, averaging 10–20 transplants per year. The first DDKT were carried out in the early 2000s. Current legislation permits living related donation (up to the fourth degree of consanguinity), spousal donation and deceased donation, while paired exchange transplantation is not yet legally allowed. In 2024, a total of 35 kidney transplants were performed (23 live related or unrelated donation transplants and 12 cadaveric kidney transplants), 2 liver transplants, 1 heart transplant, and 12 corneal transplants.

Our survey identified the lack of formal training, infrastructure, and regulatory support as the main barriers limiting the development of transplantation in Bosnia and Herzegovina. There is currently no structured clinical or surgical training program in kidney transplantation, and most healthcare professionals involved have received only short-term training abroad. Transplant teams typically include a urologist, vascular surgeon, nephrologist, anesthesiologist, immunologist, and specialized nurses. Due to staff shortages, the transplant program in Banja Luka has been suspended, leaving only Sarajevo and Tuzla as active centers.

Significant infrastructural and organizational limitations were also reported. Emerging transplant coordinators are not full-time professionals but perform coordination duties alongside routine clinical work, reducing program efficiency. While immunological testing capacity has improved, response times remain suboptimal. Standard immunosuppressive drugs are available through public funding, but access to newer agents for rejection, desensitization, and resistant infections is still limited. Legislative reform was highlighted as a top national priority, particularly the inclusion of kidney paired donation (KPD) to expand donor availability. Participants also emphasized the need for public awareness campaigns to promote organ donation, noting that current efforts led by the Donor Network of Bosnia and Herzegovina are constrained by insufficient funding.

Finally, the absence of a centralized national coordination system remains a major structural obstacle. Coordination exists only within the Federation of Bosnia and Herzegovina, which maintains a transplant waiting list, while Republika Srpska lacks a coordinator and registry. This administrative fragmentation continues to hinder national harmonization and the overall progress of transplantation in the country.

#### Kosovo

“*By adopting an official law regulating organ transplantation and building capacity, kidney transplantation can become a realistic treatment option for every eligible patient in the country*.” (Expert, Kosovo)

With 1.59 million inhabitants and a median age of 34.8 years, Kosovo has Europe’s youngest population. CKD affects approximately 11%, approximately 170,000 people. Among 1,044 patients receiving kidney replacement therapy, hemodialysis accounts for 85%–86%, around 900 patients), according to the European Renal Association registry 2022, with only 2 patients on PD. Approximately 150 patients have received transplants, with only around 8 new procedures annually, all conducted abroad.

Kosovo possesses a unique strength: a young population with strong cultural family ties. Large families are common, and relatives are willing to serve as living donors, creating a natural donor pool that could substantially increase transplantation if properly organized.

However, significant barriers prevent domestic transplantation. Kosovo lacks an officially adopted transplantation law; only a draft law exists in parliament. There is no formal surgical or clinical training in kidney transplantation and no domestic surgical team. While the nephrology team can assess transplant recipients and monitor post-transplant complications, immunological evaluation is limited with advanced testing outsourced abroad. Standard immunosuppressive drugs are publicly reimbursed, but newer agents for rejection, desensitization, and resistant infections remain article unavailable. Critically, there is no central coordination system or transplant coordinators for donor–recipient matching and follow-up.

To transform kidney transplantation into a sustainable, first-line treatment option, Kosovo must finalize and adopt comprehensive transplant legislation encompassing both paired and deceased donation. Equally important is the development of formal training programs for nephrologists, surgeons, and transplant coordinators, alongside investment in laboratory infrastructure to support advanced immunological testing. Expanding access to modern immunosuppressive therapies and establishing a centralized national transplant coordination system are also critical steps toward system-wide improvement.

#### Montenegro

“*Our legal framework exists, but it’s fragmented and not fully operational — we need harmonized regional standards*.” (Expert, Montenegro)

Montenegro, with a population of about 0.63 million and a median age of 39.7 years, has an estimated 10% CKD prevalence, roughly 6,000 individuals. Among them, 495 patients with ESKD receive renal replacement therapy, 295 patients (59.5%) undergo hemodialysis with an estimated annual incidence increase between 4%-8%, across 12 active units. On the other hand, there is currently no patient on PD. To date, around 200 kidney transplants have been performed (40.4%), with an average of 10–12 new cases conducted annually.

The legal framework for organ transplantation was introduced in 2010, and the first national legislation was enacted in 2012, marking the start of the country’s modern transplant program. That same year, the first kidney transplant in a public institution was conducted through collaboration with the Clinical Hospital Center Zagreb and the Croatian Ministry of Health. Between 2012 and 2020, this partnership enabled 47 successful LDKT and two cadaveric donations, coordinated through Eurotransplant, allowing reciprocal access for Montenegrin patients to kidney, heart, liver, and lung transplants abroad.

Currently, only living donor transplantation is performed at the Clinical Center of Montenegro in Podgorica, which serves as the country’s sole transplant facility. Their DDKT program remains inactive due to the low donor rate and limited public willingness for deceased donation. Major challenges include limited financial and infrastructural capacity, shortage of trained personnel, and regulatory gaps. There are no formal training programs in kidney transplantation, local teams collaborate with visiting surgeons from Croatia and Serbia. However, since 2019, a national HLA laboratory has been operational, ensuring basic and advanced immunological testing. Standard immunosuppressive therapies are widely available under public reimbursement schemes.

Recent legislative updates aim to strengthen the deceased donation framework, while a national public awareness campaign led by the Ministry of Health seeks to increase organ donation. The absence of a centralized transplant coordination system remains a key limitation, though efforts are ongoing to establish a national coordination network and improve donor detection and maintenance capacity in general hospitals.

#### North Macedonia

“*Even when we have trained specialists, the absence of a dedicated transplant center and staff significantly limits our transplantation services.*” (Expert, North Macedonia)

North Macedonia, located in the center of the Balkan Peninsula, has a population of approximately 1.79 million and a median age of 36.5 years. In 2024, an estimated 1,792 patients were living with ESKD, with 1,516 receiving hemodialysis and 18 on PD. Hemodialysis services, first established in 1971, are currently provided through 23 active centers. To date, 512 kidney transplants have been performed, with an average of 7–41 transplants annually.

Kidney transplantation began in 1977 with a living donor, followed by the establishment of a deceased donor program in 1987. The legal foundation, initially based on Yugoslav legislation, was updated in 2011. Despite these developments, activity remained limited for decades, averaging 7.6 and 2.9 pmp for living and deceased donation, respectively. A major turning point was heralded by the country’s integration into the Southeastern Europe Health Network (SEEHN) in 2009 and the Regional Health Development Center on Organ Donation and Transplant Medicine (RHDC) in 2011, which led to a steady rise in transplant rates, 12 pmp in 2012, 17.3 pmp in 2013, and 20 pmp in 2014 for living donors, alongside an annual 7.5 pmp for deceased donations. Activity continued to grow in subsequent years, and following the COVID-19 pandemic, a significant increase in transplantation activity was observed in both living (LD) and deceased donation (DD), with annual numbers of 8 living and 4 deceased transplants in 2020, 12 living and 16 deceased in 2021, 12 living and 10 deceased in 2022, and 14 living and 6 deceased in 2023, respectively.

In 2024, 10 kidney transplants were performed, 6 living donor and 4 deceased donor (annual 5 pmp), a temporary decline attributed to the renovation of the Urology and Transplant Departments. Additionally, 2 pediatric kidney transplants were conducted, and 282 kidney transplant recipients are currently under follow-up. The country has also expanded to other organ transplants, including liver, heart, and bone tissue procedures.

Survey responses identified several challenges: limited infrastructure, resources, and specialized training, absence of a dedicated national transplant center, and insufficient funding to sustain long- term program growth. While HLA testing and biopsy-based pathology are available, flow cytometry crossmatch remains limited to selected cases. Finally, religious and cultural beliefs continue to impede deceased donation, underscoring the need for public education and awareness campaigns to expand the donor pool and strengthen national self-sufficiency in transplantation.

#### Serbia

“*In addition to empowering national programs, fostering regional collaboration is crucial in improving transplantation services in the region*.” (Expert, Serbia)

Serbia, a country located in the central Balkans with a population of approximately 6.6 million, continues to face significant challenges in organ transplantation. It is estimated that around 700,000 people in Serbia are living with CKD. Among the 5,500 patients undergoing hemodialysis nationwide, including 40 children, only a small fraction are listed for transplantation. Despite 36 officially designated donor hospitals, only a few are functionally active, and just five centers currently perform kidney, liver, heart, and corneal transplants. The total number of transplantations remains low, averaging 17 procedures per year over the past 5 years, corresponding to a national rate of 2.5–3 PMP, one of the lowest in Europe. There are currently 832 patients in Serbia, living with a functioning kidney.

Although the Law on Transplantation of Human Organs allows both living and deceased donation, the system has been constrained by a legal vacuum following the 2021 Constitutional Court ruling that suspended the presumed consent provision (Article 23). As a result, Serbia currently operates under an explicit consent, opt-in model, requiring family authorization in all cases. Before 2010, nearly two-thirds of kidney transplants were from living donors. Since then, initiatives such as continuous brain-death monitoring have increased the number of deceased donors, but donation levels remain insufficient and inconsistent from year to year.

Despite the inclusion of paired donation in the 2018 by-law, the program has not yet been implemented, representing a missed opportunity to expand the donor pool. Persistent deficiencies in donor identification, hospital coordination, and public awareness and trust continue to limit deceased donation. Moreover, lung transplantation is not yet available in Serbia, and pediatric procedures often require the assistance of foreign surgical teams.

Strengthening training programs for transplant professionals, investing in public education, enhancing transparency and coordination, and expanding living donor options are essential steps to ensure equitable access to transplantation and to rebuild public confidence in the national system.

## Discussion

Our study found that transplantation programs in the region are small, some still in their infancy, while others despite having been established for decades remain underdeveloped. They are often characterized by low numbers, both in living and deceased donation. While all countries in the region, except Kosovo, currently perform living donor kidney transplants, activity levels remain low compared to Western Europe.

Living donation represents a significant untapped potential in the Western Balkans. European data show that living kidney donation increased modestly from 8.1 to 9.6 transplants pmp, between 2010 and 2018, though regional variation remains large, with the highest rates observed in Northern and Western Europe [12, 18]. Our findings show that in the Balkans, less than 22% of patients requiring kidney replacement therapy have received a transplant, compared to approximately 40% across other European countries [12, 19]. Surveys indicate that attitudes toward living donation in the Balkans are generally favorable, supported by strong family-oriented cultural values [20]. Moreover, given the relatively young average age of the Balkan population and the presence of established surgical and nephrology infrastructures, investing in living donor programs could significantly expand access [20]. Promoting living donation would not only strengthen national transplantation capacity but also increase the rate of preemptive kidney transplantation, aligning regional practice with European standards [13, 18, 21, 22].

Furthermore, establishing national and ultimately regional KPD programs would represent a major step toward expanding access to kidney transplantation in the Western Balkans. As shown by our findings, there are currently no active paired donation programs in the region, largely due to limited legislative frameworks, inadequate infrastructure, and insufficient resources. Even in countries such as Serbia and North Macedonia, where enabling legislation exists, KPD has not yet been implemented.

Across Europe, KPD has become a central strategy to increase LDKT rates, particularly for highly sensitized and immunologically incompatible pairs [21, 23]. Mature regional schemes, such as the Scandiatransplant STEP program, demonstrate the effectiveness of cross-matching across national boundaries, achieving higher transplantation rates and improved donor–recipient compatibility [12, 18]. Importantly, paired donation is more cost-effective than extensive desensitization protocols and avoids the clinical risks associated with highly mismatched transplants [19, 24]. Countries such as Turkey have demonstrated the feasibility and scalability of KPD even in resource-constrained settings, underscoring its relevance for the Western Balkans [12, 24, 25].

Despite progress in living donor transplantation, the absence of robust deceased donor programs remains the Achilles heel of transplantation development in the Western Balkans. Our findings revealed that deceased donation is currently performed only in North Macedonia, Serbia, and Bosnia and Herzegovina, while no active programs exist in Albania and Kosovo. In Montenegro, a deceased donor framework exists but remains largely inactive due to the very low number of donors. This stands in sharp contrast to most European countries, where deceased donation dominates the transplantation landscape [12, 26, 27].

Across Europe, recent data demonstrate a steady increase in deceased donation, including a rise in donation after circulatory death (DCD) alongside donation after brain death (DBD), driven by broader donor acceptance criteria and the widespread use of hypothermic machine perfusion [22, 23]. These advances have significantly improved graft viability and recipient outcomes [13, 28, 29]. In the latest report, the average DDKT rate rose from 21.6 to 25 pmp, between 2010 and 2018 [26]. In 2023, Council of Europe member states collectively reported 26,243 kidney transplants from 12,592 deceased donors, corresponding to a regional average of roughly 30 DDKT pmp, with leading countries such as Spain (∼60 pmp) and Croatia (∼45 pmp) among the highest performers [13, 23, 30].

However, in the Western Balkans, deceased donation remains severely limited, with an average pmp of 1–5, mainly due to the lack of trained procurement teams, insufficient infrastructure, logistics, and organ preservation technologies. Even in countries where DDKT is performed, activity levels remain far below the European average, reflecting persistent barriers such as organ shortages in North Macedonia and Serbia, and restrictive legislation in Bosnia and Herzegovina [31]. Strengthening the training of procurement professionals, supported by regional collaboration and technical assistance, is therefore essential to overcome this bottleneck and build sustainable deceased donor programs in the region.

Building on these findings, our survey identified four principal barriers currently hindering the expansion and consolidation of kidney transplantation programs in the Western Balkans.

### Formal Training and Capacity Building

Kidney transplantation training is theoretically included in both nephrology and surgical residency programs across the Western Balkans. However, the development of clinical competency is severely constrained by low procedural volume. With fewer than 25 kidney transplants performed annually in most countries, residents typically graduate having assisted with significantly fewer cases than international standards for competency, outlined by the European Union of Medical Specialists (UEMS), Division of Transplant Surgery. European Training Requirements (ETR) for Transplant Surgery [32, 33]. This low-volume exposure particularly affects surgeons and anesthesiologists, whose procedural expertise relies heavily on repetition and hands-on experience [27, 34–38]. While short-term international fellowships and online educational resources exist for transplant nephrology, they remain insufficient to build the specialized, team-based skills required to manage a high-volume program. Currently, no formal or accredited pathways for transplantation training exist in the region. This lack of specialized training has even contributed to the closure of certain centers, such as Banja Luka (Bosnia and Herzegovina).

During the May 2025 Meeting, training and capacity building were identified as priority areas. In response, ESOT experts expressed commitment to supporting the development of structured regional training programs, adapted to each country’s specific needs and current capacity level.

### Strengthening Immunological Infrastructure and Access to Modern Therapies

Our survey identified significant limitations in immunological capacity across the Western Balkans. While most centers are equipped to perform basic immunological investigations, more advanced assays, are outsourced abroad due to the lack of specialized laboratories and trained staff.

Given the small population size and limited number of transplant centers in each country, the establishment of a centralized regional immunological reference laboratory serving all Western Balkan countries would be highly beneficial. Comparable models have been successfully implemented elsewhere in Europe [28]. The Eurotransplant Reference Laboratory (ETRL) functions as a quality assurance and coordination hub for 44 tissue-typing laboratories across eight member states [39]. A similar regional HLA immunology center could provide advanced immunological testing, quality assurance and training for local laboratories, centralized crossmatching and antibody analysis protocols, and support for a future regional PKD and acceptable mismatch programs [29, 40].

In addition, participants emphasized the importance of introducing new therapeutic agents for the management of rejection, infectious viral complications, sensitized recipients and new biomarkers [41, 42]. Wider access to novel immunosuppressive drugs and biologics, was identified as a regional priority to align treatment standards with those applied in leading European centers [13, 32, 33].

### Developing Effective Coordination and Governance in Transplantation

Our survey revealed that Albania, Bosnia and Herzegovina, Montenegro and Kosovo, lack a centralized coordination mechanism and dedicated transplant coordinators, leading to fragmented communication between institutions, suboptimal donor identification, and prolonged waiting times for transplant candidates. Although North Macedonia and Serbia have established national coordination structures, both require more sustainable training and improved operational frameworks to function effectively.

Strengthening coordination at both national and inter-hospital levels is therefore a critical priority. The Croatian model provides a well-documented example of success. Following the introduction of a fully integrated national coordination system, including a central registry, standardized donor management protocols, and dedicated transplant coordinators, Croatia’s deceased donor rate rose sharply to 33–36 per million population (pmp), surpassing most EU countries [43–45]. This transformation established Croatia among Europe’s top-performing countries in organ donation and transplantation [46].

Adopting similar frameworks across the Western Balkans could optimize donor identification, ensure equitable organ allocation, and promote transparency and accountability at both the national and regional levels. Such systems would also provide a foundation for future cross-border collaboration and the gradual harmonization of donation and transplantation practices within the region.

### Addressing Legislative and Cultural Barriers to Transplantation

Our findings reveal a fragmented legislative landscape that continues to constrain kidney transplantation across the Western Balkans. Kosovo currently lacks comprehensive transplantation legislation, with only a draft law under development, while Albania has no specific framework regulating deceased organ donation. The remaining four countries, Serbia, North Macedonia, Montenegro, and Bosnia and Herzegovina have enacted legislation permitting both living and deceased donation, yet implementation remains inconsistent and largely inadequate. Although paired donation is legally permitted in some countries, no national program has yet been established in the region. In contrast, EU member states with robust legislative frameworks, demonstrate how clear legal definitions of consent systems, centralized coordination, and continuous professional training can yield deceased donation rates exceeding 35–60 pmp, compared to approximately 1 – 5 pmp in most Western Balkan countries [13, 26, 43, 47]. Beyond legislative gaps, significant cultural and religious beliefs restrict deceased donation, particularly where public awareness remains low and mistrust toward healthcare institutions persists. Effective reform should therefore combine legislative modernization, with the establishment of active patient organizations emphasizing the advantages of kidney transplantation within the ESKD patient community, as well as participating in broad education and public awareness campaigns involving religious leaders, media, stakeholders, policymakers and civil society organizations to build trust and promote organ donation [48, 49].

### Strengthening Regional Integration in Transplantation

During the May 2025 Meeting, participants emphasized the need to strengthen regional collaboration in kidney transplantation, given the low procedural volumes and shared healthcare challenges across the Western Balkans. Establishing a regional network to facilitate experience exchange, collaboration on complex cases, and organize short-term, hands-on training programs was identified as a critical first step toward expanding national capacities [50, 51]. A successful precedent exists in North Macedonia, where transplantation activity increased following integration into the Southeastern Europe Health Network (SEEHN) and the Regional Health Development Center on Organ Donation and Transplant Medicine (RHDC). Building on this model, participants proposed annual regional meetings to review progress, harmonize practices, and address program-specific challenges [16, 52]. In the long term, implementing standardized clinical protocols and a regional KPD program, modeled after the Turkish and Scandiatransplant (STEP) systems, could significantly expand the donor pool and improve compatibility for highly sensitized patients [13, 31, 53]. In light of the current transplantation landscape across the Western Balkans, consistent with the findings of our survey, ESOT in collaboration with the WHO, launched in September 2025 an Operational and Technical Guidance for Developing a Transplant Programme in the Balkans. This comprehensive framework provides a roadmap for establishing larger, more efficient, and self- sustaining transplantation programs in the region [54].

## Conclusion

This project represents the first comprehensive collection of real-world data and expert insights on the major challenges facing kidney transplantation in the Western Balkans. During this process, certain obstacles were identified toward a solid and sustainable kidney transplant program in the region. Fortunately, all of them can be overcome, especially in the context of EU membership to which all of Western Balkans avidly aspire.

We hope this initiative further solidified by the inspiring meeting in Tirana in May 2025, can serve as a steppingstone for regional progress guiding the development of realistic and practical solutions to address identified barriers and foster long-term growth and sustainability of transplantation services throughout the region. It is our mission to offer all our patients timely and equitable access to state- of-the-art transplant procedures in the Western Balkans. It will not be easy, but it will take more than that to stop us.

## Data Availability

The original contributions presented in the study are included in the article/Supplementary Material, further inquiries can be directed to the corresponding author.
