# Supplementary material for: Kidney Transplantation in Western Balkans: A Regional Blueprint for Access, Capacity, and Equity
Source: Transpl Int. 2026 Mar 13;39:15952. doi: 10.3389/ti.2026.15952 (PMC13023137; doi:10.3389/ti.2026.15952)
Supplement: Supplementary file 1 [file Table1.docx]

Supplementary Material

# Supplementary Figures and Tables

| **Thematic Domain** | **Delphi Statement** | **Delphi Round** | **Agreement (%)** | **Consensus Outcome** |
| --- | --- | --- | --- | --- |
| **Training & Capacity Building** | There is an insufficient number of formally trained transplant surgeons and nephrologists in the region. | Round 1 | 88% | Accepted |
|  | Structured national and regional training programs in transplantation should be developed. | Round 2 | 92% | Accepted |
|  | Dedicated training based on individual country needs should be developed. | Round 2 | 95% | Accepted |
| **Infrastructure & Immunological Capacity** | Limited access to advanced immunological testing limits transplantation activity. | Round 1 | 78% | Accepted |
|  | Establishment of centralized or shared regional immunological laboratories would improve transplant capacity. | Round 2 | 80% | Accepted |
|  | Investing in expanding existing transplant infrastructure. | Round 2 | 97% | Accepted |
| **Coordination & Data Systems** | Establishment of national transplant coordination bodies and improving current ones, is necessary for system strengthening. | Round 1 | 92% | Accepted |
|  | Absence of national transplant registries limits planning and quality monitoring. | Round 2 | 87% | Accepted |
| **Legislative & Regulatory Framework** | Implementing region-wide legislative reforms to align legal frameworks across Western Balkans. | Round 1 | 93% | Accepted |
|  | Paired kidney exchange should be formally included in national transplant legislation in countries where it is currently lacking. | Round 1 | 98% | Accepted |
|  | Introduction of legal provisions for deceased donor kidney transplantation in countries that don’t perform it, is a regional priority. | Round 2 | 80% | Accepted |
|  | Cultural and religious barriers represent a significant limitation to deceased donation programs. | Round 2 | 100% | Accepted |
| **Regional Collaboration** | Establishing a regional paired donation program would significantly expand the donor pool. | Round 1 | 100% | Accepted |
|  | A regional exchange program would ensure better access, more equitable organ allocation and improved transparency. | Round 2 | 95% | Accepted |
|  | Develop a regional collaboration network to promote experience and knowledge sharing. | Round 2 | 100% | Accepted |
| *Consensus was defined a priori as ≥75% agreement among participating experts. Statements reaching this threshold were retained in the final framework, while those below the threshold were excluded or revised.* | | | | |

**Supplementary Table 1.** Summary of Delphi Statements, Expert Agreement, and Consensus Outcomes
